# Ezetimibe ameliorates atherogenic lipids profiles, insulin resistance and hepatocyte growth factor in obese patients with hypercholesterolemia

**DOI:** 10.1186/1476-511X-14-1

**Published:** 2015-01-10

**Authors:** Hisashi Adachi, Hitoshi Nakano, Kiichiro Yamamoto, Masashi Nakata, Hisatoshi Bekki, Tomoki Honma, Hideki Yoshiyama, Masatoshi Nohara, for the ERASE METS Study Investigators

**Affiliations:** Department of Community Medicine, Kurume University School of Medicine, 67 Asahi-machi, Kurume, 830-0011 Japan; Nakano Medical Clinic, Chikugo, Japan; Yamamoto Clinic, Chikugo, Japan; Nakata Clinic, Kurume, Japan; Bekki Clinic, Yame, Japan; Honma Clinic, Kurume, Japan; Yoshiyama Medical Clinic, Chikugo, Japan; Nohara Clinic, Kurume, Japan

**Keywords:** Anti-obesity agents, Metabolic syndrome, Insulin, Hypolipidemic agents

## Abstract

**Background:**

Ezetimibe ameliorates serum low-density lipoprotein cholesterol (LDL-c) and it has been approved for the treatment of hypercholesterolemia. However, the effects of ezetimibe on specific biomarkers have not been fully clarified in obese Japanese patients.

**Methods:**

A total of 101 patients (47 males and 54 females) were registered in this study during 2011 and 2012. All patients were over 20 years old, were obese [body mass index (BMI) ≥ 25kg/m^2^] and had hypercholesterolemia (LDL-c ≥ 120mg/dl). After excluding 10 subjects (7 who dropped out and 3 with missing data), 91 patients (39 males and 52 females) were analyzed. They were treated with 10 mg ezetimibe once a day for 24 weeks and were evaluated at 12 and 24 weeks.

**Results:**

Following 12 weeks of ezetimibe therapy, BMI (p < 0.001), waist circumference (p < 0.001), total cholesterol (p < 0.001), LDL-c (p < 0.001), non high-density lipoprotein cholesterol [HDL-c] (p < 0.001), triglycerides (p < 0.05) and remnant-like particle cholesterol (RLP-c; p < 0.001) were significantly decreased. Following 24 weeks of ezetimibe therapy, BMI (p < 0.05), waist circumference (p < 0.001), total cholesterol (p < 0.001), LDL-c (p < 0.001), non HDL-c (p < 0.001), triglycerides (p < 0.05), RLP-c (p < 0.001), insulin (p < 0.05) and hepatocyte growth factor (HGF; p < 0.05) were significantly decreased. In contrast, HDL-c (p < 0.001) was significantly increased.

**Conclusions:**

Ezetimibe ameliorated not only atherogenic lipid profiles but also anthropometric factors, insulin resistance and biomarkers such as HGF. Ezetimibe may have pleiotropic effects on obese patients with hypercholesterolemia.

## Background

Ezetimibe is a novel lipid-lowering agent that inhibits intestinal absorption of dietary and biliary cholesterol. The effects of ezetimibe on low-density lipoprotein cholesterol (LDL-c), as well as on baseline lipid profile, hypertension, diabetes mellitus and body mass index (BMI) were found to be generally consistent across all subgroups analyzed [1]. Numerous clinical studies [2–10] have revealed that combination therapy with ezetimibe is beneficial in reducing LDL-c, remnant-like particle cholesterol (RLP-c), and triglycerides, and in increasing high-density lipoprotein cholesterol (HDL-c) in patients with metabolic syndrome (MetS) or diabetes. Ezetimibe may have more effect in patients with MetS than in non-obese subjects [11]. We and others clarified that the values of RLP-c, aldosterone and hepatocyte growth factor (HGF) were elevated in patients with MetS [12–17]. Moreover, the levels of adiponectine and the ratio of eicosapentaenoic acid to arachidonic acid (EPA/AA) were decreased in patients with MetS [18–20]. The purpose of the present study was to investigate whether the levels of such biomarkers were improved by ezetimibe either in monotherapy or combined therapy in Japanese obese subjects [21].

## Results

Table 1 shows the characteristics of the 101 subjects (47 males and 54 females). The mean age of the subjects was 56.7 ± 11.3 years in males and 63.1 ± 11.6 years in females. The mean BMI was 27.7 ± 3.6kg/m^2^ in males and 27.6 ± 3.4kg/m^2^ in females. The mean waist circumference was 94.6 ± 10.4cm in males and 95.7 ± 8.1cm in females. As apparent from Table 1, the prevalence of metabolic syndrome-related factors was high. The mean height (p < 0.001) and weight (p < 0.001) were significantly higher in males than in females. The mean age (p < 0.01), total cholesterol (p < 0.01), LDL-c (p < 0.05), HDL-c (p < 0.05), non HDL-c (p < 0.05) and adiponectine (p < 0.01) were significantly lower in males than in females.

**Table 1:** **Characteristics of patients**

Characteristics	Males (n = 47)	Females (n = 54)	p
Age (years)	56.7 ± 11.3	63.1 ± 11.6	0.008
Height (cm)	166.9 ± 5.9	152.9 ± 8.0	<0.001
Weight (kg)	77.3 ± 12.7	64.6 ± 10.1	<0.001
Body mass index (kg/m^2^)	27.7 ± 3.6	27.6 ± 3.4	0.919
Waist circumference (cm)	94.6 ± 10.4	95.7 ± 8.1	0.622
Systolic blood pressure (mmHg)	129.1 ± 14.3	127.9 ± 15.4	0.705
Diastolic blood pressure (mmHg)	77.1 ± 8.5	74.7 ± 10.4	0.229
Total cholesterol (mg/dl)	223.3 ± 32.6	243.6.1 ± 35.1	0.003
LDL-cholesterol (mg/dl)	138.7 ± 26.9	152.2 ± 29.7	0.020
HDL-cholesterol (mg/dl)	48.8 ± 10.1	53.6 ± 10.7	0.024
Non HDL-cholesterol (mg/dl)	174.5 ± 29.8	190.1 ± 31.1	0.012
Triglycerides* (mg/dl)	172.4 (56–1804)	151.4 (62–473)	0.214
RLP-cholesterol* (mg/dl)	6.7 (2.2-82.5)	6.5 (2.4-28.7)	0.868
Free fatty acid* (mEq/l)	428.3 (92–1423)	437.0 (87–1134)	0.834
Plasma glucose (mg/dl)	108.5 ± 26.7	103.4 ± 29.1	0.360
Insulin* (μU/ml)	12.9 (1.55-200)	10.2 (2.82-72.6)	0.180
HOMA index*	3.36 (0.35-80.5)	2.51 (0.64-36.2)	0.146
hs CRP* (mg/dl)	720.5 (50-19100)	713.4 (50-7910)	0.990
Aldosterone (pg/dl)*	95.6 (42.2-315.0)	85.6 (37.9-296.0)	0.238
HGF (ng/ml)	0.32 ± 0.05	0.31 ± 0.06	0.731
Adiponectine (μg/ml)*	1.97 (0.28-10.6)	3.21 (0.30-8.34)	0.001
EPA/AA*	0.35 (0.10-2.05)	0.30 (0.10-1.59)	0.226

Data are means (SD), geometric mean and range. *: These variables are shown in the original scale after analysis using log (natural)-transformed values.

*Abbreviations*: *RLP* Remnant-like particle cholesterol, *HOMA* Homeostasis model assessment, *HGF* Hepatocyte growth factor, *EPA/AA* Eicosapentaenoic acid to arachidonic acid.

Table 2 shows the anthropometric and laboratory data before, at 12 weeks, and at 24 weeks after ezetimibe treatments. Following 12 weeks of ezetimibe therapy, BMI (p < 0.001), waist circumference (p < 0.001), total cholesterol (p < 0.001), LDL-c (p < 0.001), non HDL-c (p < 0.001), triglycerides (p < 0.05) and RLP-c (p < 0.001) were significantly decreased. Following 24 weeks of ezetimibe therapy, BMI (p < 0.05), waist circumference (p < 0.001), total cholesterol (p < 0.001), LDL-c (p < 0.001), non HDL-c (p < 0.001), triglycerides (p < 0.05), RLP-c (p < 0.001), insulin (p < 0.05) and HGF (p < 0.05) were significantly decreased. In contrast, HDL-c (p < 0.001) was significantly increased. There were no significant changes in systolic and diastolic blood pressure (BP), free fatty acid (FFA), plasma glucose, homeostasis model assessment (HOMA) index, high sensitive CRP (hsCRP), aldosterone, adiponectine or EPA/AA.

**Table 2:** **Lipids and other biomarkers at baseline and at 3 and 6 months after the ezetimibe therapy**

Parameters	Pre	Post 3M	Post 6M
Body mass index (kg/m^2^)	27.9 ± 3.5	27.5 ± 3.4***	27.6 ± 3.4*
Waist circumference (cm)	93.6 ± 8.5	91.8 ± 7.8***	91.5 ± 7.9***
Systolic blood pressure (mmHg)	128.7 ± 14.8	127.2 ± 13.9	127.0 ± 13.0
Diastolic blood pressure (mmHg)	76.0 ± 9.4	75.6 ± 9.4	74.4 ± 10.3
Total cholesterol (mg/dl)	236.2 ± 34.7	208.9 ± 31.9***	207.0 ± 34.5***
LDL-cholesterol (mg/dl)	147.9 ± 29.5	125.5 ± 25.9***	126.5 ± 30.5***
HDL-cholesterol (mg/dl)	51.7 ± 10.7	52.7 ± 10.3	54.5 ± 9.8***
Non HDL-cholesterol (mg/dl)	184.5 ± 30.7	156.2 ± 29.1***	151.9 ± 32.6***
Triglycerides^+^ (mg/dl)	162.4	145.5*	134.3***
RLP-cholesterol^+^ (mg/dl)	6.6	5.2***	4.7***
Free fatty acid^+^ (mEq/l)	437.0	454.8	483.0
Plasma glucose (mg/dl)	106.4 ± 28.8	107.3 ± 24.5	103.3 ± 24.1
Insulin^+^ (μU/ml)	11.9	11.1	10.0*
HOMA index^+^	3.1	2.8	2.6
hsCRP^+^ (mg/dl)	765.1	820.6	871.3
Aldosterone (pg/dl)^+^	90.9	97.5	96.5
HGF (ng/ml)	0.32 ± 0.06	0.31 ± 0.04	0.30 ± 0.03*
Adiponectine (μg**/**ml)^+^	2.58	2.64	2.53
EPA/AA^+^	0.31	0.31	0.31

*: p < 0.05, ***: p < 0.001.

Data are means (SD), geometric mean and range. *: These variables are shown in the original scale after analysis using log (natural)-transformed values.

*Abbreviations*: *RLP* Remnant-like particle cholesterol, *HOMA* Homeostasis model assessment, *HGF* Hepatocyte growth factor, *EPA/AA* Eicosapentaenoic acid to arachidonic acid.

Table 3 shows the anthropometric and laboratory data before and at 24 weeks after ezetimibe monotherapy or combined therapy. Following 24 weeks of ezetimibe monotherapy, BMI (p < 0.05), waist circumference (p < 0.001), systolic (p < 0.05) and diastolic (p < 0.05) BP, total cholesterol (p < 0.001), LDL-c (p < 0.001), non HDL-c (p < 0.001), triglycerides (p < 0.05), RLP-c (p < 0.01), plasma glucose (p < 0.05), insulin (p < 0.01), HOMA index (p < 0.01) and HGF (p < 0.05) were significantly decreased. In contrast, HDL-c (p < 0.001) was significantly increased. Following 24 weeks of combined therapy, BMI (p < 0.05), waist circumference (p < 0.01), total cholesterol (p < 0.05), LDL-c (p < 0.05), non HDL-c (p < 0.01), triglycerides (p < 0.001) and RLP-c (p < 0.001) were significantly decreased. In contrast, HDL-c (p < 0.001) was significantly increased.

**Table 3:** **Laboratory findings before and after ezetimibe monotherapy and combined therapy**

	Monotherapy	(n = 71)	Combined therapy	(n = 20)
Parameters	Pre	Post 6M	Pre	Post 6M
Body mass index (kg/m^2^)	28.2 ± 3.7	28.0 ± 3.8	27.2 ± 2.0	25.9 ± 1.6*
Waist circumference (cm)	94.6 ± 8.2	92.8 ± 8.6***	91.0 ± 5.6	88.3 ± 5.3**
Systolic blood pressure (mmHg)	130.1 ± 14.6	128.8 ± 13.2*	125.6 ± 13.8	121.8 ± 11.2
Diastolic blood pressure (mmHg)	77.3 ± 9.5	75.9 ± 10.6*	71.7 ± 8.2	69.5 ± 7.7
Total cholesterol (mg/dl)	237.9 ± 35.9	212.0 ± 33.0***	224.7 ± 27.8	192.2 ± 36.1*
LDL-cholesterol (mg/dl)	151.3 ± 28.6	132.4 ± 28.6***	132.1 ± 26.8	107.8 ± 30.5*
HDL-cholesterol (mg/dl)	51.9 ± 9.9	55.1 ± 9.4***	50.8 ± 11.9	53.3 ± 11.5***
Non HDL-cholesterol (mg/dl)	184.9 ± 31.7	155.6 ± 32.5***	174.6 ± 25.8	141.8 ± 32.2**
Triglycerides^+^ (mg/dl)	157.5	129.2***	210.9	155.3***
RLP-cholesterol^+^ (mg/dl)	6.3	4.7**	8.2	4.7***
Free fatty acid^+^ (mEq/l)	435.9	505.6*	435.4	429.7
Plasma glucose (mg/dl)	105.1 ± 27.9	102.6 ± 26.4*	109.3 ± 21.7	105.1 ± 15.9
Insulin^+^ (μU/ml)	12.1	9.6**	11.7	11.4
HOMA index^+^	3.0	2.4**	3.2	2.9
hsCRP^+^ (mg/dl)	807.0	883.7*	686.1	879.6*
Aldosterone (pg/dl)^+^	86.9	92.9*	101.6	110.8
HGF (ng/ml)	0.33 ± 0.07	0.31 ± 0.04*	0.31 ± 0.03	0.30 ± 0.02
Adiponectine (μg**/**ml)^+^	2.51	2.56	2.49	2.47
EPA/AA^+^	0.29	0.28	0.39	0.40

*: p < 0.05, **: p < 0.01, ***: p < 0.001.

Data are means (SD), geometric mean and range. *: These variables are shown in the original scale after analysis using log (natural)-transformed values.

*Abbreviations*: *RLP* Remnant-like particle cholesterol, *HOMA* Homeostasis model assessment, *HGF* Hepatocyte growth factor, *EPA/AA* Eicosapentaenoic acid to arachidonic acid.

## Discussion

Twenty-four weeks of ezetimibe treatment ameliorated not only atherogenic lipid profiles but also anthropometric factors, insulin resistance and biomarkers such as HGF. To the best of our knowledge, this is the first report to show that ezetimibe treatment reduced HGF levels in obese patients with hypercholesterolemia.

Although many studies [3–10] have reported that ezetimibe treatment ameliorated atherogenic lipid profiles, very few [22, 23] have referred to anthropometric data. One report from Japan [22] found no significant differences in body weight and BMI after ezetimibe treatment. Other Japanese investigators [24] also suggested that there were no significant changes in metabolic markers including BMI and waist circumference before and after ezetimibe treatment. Although Yagi S, et al. [23] demonstrated that ezetimibe treatment significantly reduced body weight, BMI, waist circumference and BP, they did not discuss the reason. Our data were consistent with theirs in large part. One possible explanation is that ezetimibe markedly reduced visceral fat as assessed by abdominal computed tomography [25]. Our data and others [23–25] suggested that ezetimibe may play a unique role in the treatment of metabolic syndrome. The present study revealed that ezetimibe treatment also ameliorated insulin resistance in addition to lipid profiles. In one experimental study, ezetimibe was shown to improve insulin resistance in Zucker fatty rats, a model of obesity [26].

We confirmed that ezetimibe improves HOMA index as a marker of insulin resistance. Dagli N, et al. [27] reported that low-dose statin (pravastatin) and ezetimibe combination therapy improved insulin resistance markedly better than high-dose pravastatin monotherapy. This suggested that combined antilipidemic therapy may be a more favorable treatment alternative in high-risk dyslipidemic patients. Hiramitsu S, et al. [24] also demonstrated that ezetimibe significantly reduced the fasting insulin level (-12.8% reduction) and glycosylated hemoglobin A_1c_ [HbA_1c_] (-3.4% reduction). However, Kikuchi K, et al. [28] reported that ezetimibe restored the postprandial dysregulation of lipid but did not affect glucose metabolism in a double-blind randomized crossover trial.

Interestingly, ezetimibe monotherapy was more effective than combined therapy in reducing insulin and HOMA index in this study. These results may be consistent with our recent report [29].

We have shown that HGF levels as well as RLP-c were significantly reduced by twenty-four weeks of ezetimibe treatment. Our colleagues [16] demonstrated that HGF levels were significantly and strongly associated with metabolic syndrome. HGF is one of the adipocytokines. A report [30] described the relationship between obesity and serum HGF levels. It may be feasible to consider that the elevated HGF levels in obese subjects may be due to fatty liver secondary to obesity. It is interesting to note that results of the present study using ezetimibe only (n = 83) are consistent with a previous report [13]. Taken together, candidate subjects suitable for ezetimibe monotherapy may have multiple syndromes with underlying insulin resistance.

A limitation of our study is that it was an observational study without a control group. This study design did not allow us to evaluate the impact of ezetimibe on insulin resistance over time. In this study population, we also recommended standard diet and exercise therapies. To exclude the effects of the standard diet and exercise therapies, we examined additional data from another cohort. We enrolled 60 subjects, who received health check-up examinations in 2001 and 2003 in Uku town in Japan, who took no medicine, and who were recommended standard diet and exercise therapies. Their HOMA-IR was 1.21 ± 0.84 at baseline in 2001 and 1.31 ± 0.87 at 2-year follow-up in 2003. This group showed no significant change in insulin resistance (p = 0.272), suggesting that standard diet and exercise therapies have no significant effects on insulin resistance. Second, this study was conducted in Japan, where the incidence of obesity is low compared with Caucasians. A final limitation is that the number of participants and the follow-up period may have been insufficient to fully elucidate the role of ezetimibe. Nevertheless, the pleiotropic effects of the ezetimibe treatment were striking and deserve further investigation. Additional studies of ezetimibe with a large sample size including predictive measures of major atherosclerotic diseases are needed.

## Conclusions

In conclusion, ezetimibe ameliorated not only atherogenic lipid profiles but also anthropometric factors, insulin resistance and biomarkers such as HGF. Ezetimibe may have pleiotropic effects on obese patients with hypercholesterolemia.

## Methods

### Ethical statement

This study was approved by the Institutional Review Board of Community Medicine, Kurume University School of Medicine, as well as by the ethics committee of Kurume University. All subjects participated in the study after making signed informed consents.

### Study population and data collection

All outpatients aged over 20 years with obesity (BMI ≥ 25kg/m^2^) and hypercholesterolemia (LDL-c ≥ 120mg/dl) were registered in this study at Kurume University Hospital or nearby clinics during 2011 and 2012. After excluding 10 subjects (7 who dropped out and 3 with missing data), 91 patients (39 males and 52 females) were analyzed.

The study was named ERASE METS (*E*ffects on *R*egression of *A*thero*S*clerotic risks by *E*zetimibe for the patients with *MET*abolic *S*yndrome) by one of the study investigators (T.H.). The enrolled subjects were treated with 10 mg ezetimibe once a day for 24 weeks and were evaluated at 12 and 24 weeks. We divided the patients into two groups, one without anti-dyslipidemic agents (ezetimibe monotherapy, n = 71) and the other with a statin (combined therapy, n = 20). Standard diet and exercise therapy for dyslipidemia were recommended during the study. Height was measured with shoes on and weight was measured with the patients in their ordinary clothing. BMI was calculated as weight (kilograms) divided by the square of height (square meters) as an index of obesity. In Japan, obesity is defined as BMI over 25kg/m^2^. Waist circumference was measured at the level of the umbilicus in the standing position. BP was measured in the supine position twice at 3-min intervals using an upright standard sphygmomanometer. Vigorous physical activity and smoking were avoided for at least 30 min before BP measurement. The second BP with the fifth-phase diastolic pressure was used for analysis. Blood was drawn from the antecubital vein in the morning after a 12-hour fast for determinations of lipids profiles [total cholesterol, LDL-c, triglycerides, HDL-c, non-HDL-c and RLP-c], free fatty acid (FFA), fasting plasma glucose (FPG), fasting immune-reactive insulin (IRI), high-sensitivity C-reactive protein (hs-CRP), aldosterone, HGF, adiponectine and EPA/AA. Fasting blood samples were centrifuged within 1 hour after collection. The serum levels of total cholesterol, LDL-c, triglycerides, HDL-c and non-HDL-c, FFA, FPG and IRI were measured using standard laboratory methods. Serum RLP-c was measured by immune-separation technique [31] (using an immune-affinity gel containing monoclonal antibodies to human apolipoprotein [apo] B-100 and apo A-1). HOMA index was calculated as FPG (mg/dL) × fasting IRI (μU/mL)/405 and used as a marker of insulin resistance [32]. Hs-CRP was measured as an inflammatory marker. Plasma aldosterone was measured in the morning by radioimmunoassay (RIA), and samples were taken after the subjects had remained in a sitting position for 10 minutes [14]. Plasma HGF levels were measured by the enzyme-linked immunosorbent assay (ELISA) [33]. Serum adiponectin concentrations were measured by radioimmunoassay [18]. Measurement of serum EPA/AA levels were outsourced to SRL (Fukuoka, Japan) [20]. All of the blood tests except for HGF, adiponectin and EPA/AA were measured in a commercially available laboratory (The Kyodo Igaku Laboratory, Fukuoka, Japan).

### Statistical analysis

Results are presented as the mean ± standard deviation. Variables that were not normally distributed and/or displayed homogeneity of variances were analyzed by Mann–Whitney test for independent samples. Normally distributed variables were analyzed using a paired *t* test. The p-values < 0.05 were considered statistically significant. All statistical analyses were performed using SAS version 9.3 (SAS Inc., Cary, NC, USA).

## Abbreviations

LDL-c: Low-density lipoprotein cholesterol
BMI: Body mass index
HDL: High-density lipoprotein
RLP-c: Remnant-like particle cholesterol
HGF: Hepatocyte growth factor
MetS: Metabolic syndrome
EPA: Eicosapentaenoic acid
AA: Arachidonic acid
BP: Blood pressure
HOMA: Homeostasis model assessment
HbA_1c_: Glycosylated hemoglobin A_1c_
FFA: Free fatty acid
FPG: Fasting plasma glucose
IRI: Immune-reactive insulin
Hs-CRP: High-sensitivity C-reactive protein
RIA: Radioimmunoassay
ELISA: Enzyme-linked immunosorbent assay.
